# Exploring the impact of Namaste Care for individuals with advanced dementia: a systematic review of costs, effects and benefits

**DOI:** 10.1136/bmjopen-2025-102899

**Published:** 2026-05-06

**Authors:** Tereza Parks, Jana Soukopová, Dominika Tóthová, Hanneke J A Smaling, Aileen Murphy, Lucie Vidovićová, Jakub Hlavka

**Affiliations:** 1 Masaryk University, Brno, Czech Republic; 2 Public Health and Primary Care, Leiden Universitair Medisch Centrum, Leiden, The Netherlands; 3 University Network of the Care Sector South Holland, Leiden University Medical Center, Leiden, The Netherlands; 4 Economics, University College Cork, Cork, Ireland

**Keywords:** Treatment Outcome, Dementia, Health Care Costs, Quality of Life

## Abstract

**Objectives:**

Namaste Care, a non-pharmaceutical daily multicomponent palliative care intervention, offers care for people with dementia, aiming to improve quality of life of those living with dementia as well as their family and caregivers. This systematic review explores the Namaste Care intervention and its clinical and economic effects in multiple care settings. The aim of this review is to consolidate existing evidence on Namaste Care’s clinical and economic outcomes and examine the tools used for data collection.

**Design:**

A systematic literature search was conducted (PubMed, Scopus and Web of Science) to identify peer-reviewed studies on Namaste Care’s impact on quality of life, costs, health, economic outcomes and benefits up to 22 February 2026. Methodological quality was assessed using the Mixed Methods Appraisal Tool, while the completeness of reporting of economic evaluation studies was evaluated according to the Consolidated Health Economic Evaluation Reporting Standards 2022 (CHEERS).

**Results:**

31 studies reported the clinical and/or economic outcomes of Namaste Care. The results for quality of life and quality of dying were mixed, while 5 of 11 studies evaluating quality of life reported significant improvements. The various quality-of-life instruments used include the Quality of Life in Late-Stage Dementia (QUALID), EQ-5D-3L and EQ-5D-5L instruments, ICEpop CAPability Measure for Older People (ICECAP-O), ICECAP Supportive Care Measure (ICECAP-SCM), Quality of Life for People with Dementia (QUALIDEM) and Carers-DEMentia Quality of Life (C-DEMQOL). The clinical outcomes considered included pain, behavioural symptoms and quality of end-of-life care. The Medication Quantification Scale and Minimum Data Set indicated reductions in antidepressant and antianxiety medication use. Seven studies reported significant improvements in well-being, and two studies reported reduced stress among family members following Namaste Care sessions. A subset of five studies reported a range of economic outcomes.

**Conclusion:**

The findings suggest that Namaste Care improves well-being, reduces caregiver stress and lowers the use of antidepressant and antianxiety medications at a moderate cost. The current literature is characterised by small, non-random, heterogeneous studies. Randomised controlled trials, which include economic evaluations, help to improve evidence-based research to support funding and implementation decisions on Namaste Care.

**PROSPERO registration number:**

CRD42024560056.

**STRENGTHS AND LIMITATIONS OF THE STUDY**

- A comprehensive search strategy, including database searches and citation snowballing, was used to ensure broad and inclusive coverage of relevant studies.
- All eligible studies were included regardless of methodological quality, allowing for a diverse and representative overview of existing evidence.
- A structured quality assessment was conducted to aid in the interpretation of findings and to provide transparency regarding the strength of the evidence base.
- The review was limited to peer-reviewed, English-language publications that explicitly referenced Namaste Care, which may have excluded relevant studies published in other languages or under different terminology.
- The short duration of the included studies restricts the ability to draw conclusions about the long-term effectiveness and cost implications of the intervention for individuals with dementia, caregivers and care staff.

## Introduction

 Over 57 million individuals worldwide live with dementia, a syndrome characterised by progressive cognitive and functional impairment that is most commonly caused by Alzheimer’s disease and other neurodegenerative and cerebrovascular disorders. With nearly 10 million new cases reported annually^1^ and the older population at risk, protection of quality of life (QoL) is imperative. End-of-life care for people living with dementia is especially vital, as there is no cure for dementia.

Various care options and programmes are being developed to improve QoL in patients with dementia. Namaste Care is one such approach. Namaste Care is a non-pharmaceutical intervention used in nursing homes and other settings in countries around the world with the objective of increasing the QoL of people living with dementia as well as their family members and caregivers.^2^ It derives its name from the Hindi greeting ‘to honor the spirit within’, which emphasises the dignified transition of people with dementia as they are near the end of life.^3 4^ A unique aspect of this care regimen is the option for individual sessions within designated spaces known as ‘Namaste rooms’^5^,^7^ or ‘sensory rooms’, which are characterised by elements such as soft lighting, tranquil music and pleasing fragrances to foster a calming ambiance.^8^ Alternatively, Namaste sessions may occur within the resident’s room or in communal areas arranged as ‘salons’ with chairs arranged in a circular configuration.^9^ Additionally, Namaste Care may extend to home settings for individuals cared for by a family member or caregiver.^10^,^14^ Ideally, Namaste Care should be administered daily with morning and evening sessions, each lasting 2 hours.^5 15^

The Namaste Care sessions involve creating soothing environments and incorporating physical touch, music and other sensory activities to stimulate the senses. Familiar objects and mementos are included, along with the availability of snacks and drinks, to encourage hydration and foster positive taste experiences.^15^ Caregivers often engage participants through gentle hand massage, aromatherapy and extra personal care. The programme uses a person-centred approach in which activities are used to meaningfully connect with the person with dementia. By addressing the emotional, social and physical well-being of participants, Namaste Care has shown promise in reducing symptoms of agitation while promoting relaxation and enhancing overall QoL for those with late-stage dementia.^4^

Namaste Care, like any specialised service, is resource-intensive and requires thoughtful evaluation of its efficiency. While cost-effectiveness may not be the primary objective, considering costs is vital for maintaining long-term sustainability. Previous studies have explored select benefits of Namaste Care, but no systematic review of its clinical and economic effects has been conducted to date. We address this gap in the available literature. The primary aim of this review is to consolidate existing evidence of Namaste Care’s clinical and economic outcomes and to comprehensively examine the tools used to gather data. Specifically, the review synthesises the intervention’s outcomes concerning individuals with dementia, their caregivers and staff, focusing on Namaste Care’s impact on QoL, symptom management, caregiver well-being and economic outcomes.

## Methods

### Protocol

The protocol is registered with the International Prospective Register of Systematic Reviews (PROSPERO; registration number CRD42024560056). We report our findings in accordance with the Preferred Reporting Items for Systematic Reviews and Meta-Analyses (PRISMA) statement^16^ and the COnsensus-based Standards for the selection of health Measurement INstruments (PRISMA-COSMIN) for Outcome Measurement Instruments.^17^

### Search strategy

A systematic literature search was performed in PubMed, Scopus and Web of Science databases between 21 June 2024 and 22 February 2026. In all the databases, terms related to ‘Namaste Care’, ‘Cost’, ‘Benefit’, ‘Effect’, ‘Effectiveness’, ‘Outcome’ and ‘Quality of life’ were combined (table 1). Additional records were identified via manual searches using backward and forward snowballing.

**Table 1:** Systematic review search—search terms and filters used during the systematic review search

Database	Search field	Search string
Web of Science	Topic (Title, Abstract, Keywords)	TS=(“Namaste Care”) AND TS=(cost OR benefits OR effectiveness OR outcome OR “quality of life”)
Scopus	Title, Abstract, Keywords	TITLE-ABS-KEY(“Namaste Care”) AND TITLE-ABS-KEY(cost OR benefits OR effectiveness OR outcome OR “quality of life”)
PubMed	Title, Abstract	(“Namaste Care”(Title/Abstract)) AND (“cost”(Title/Abstract)OR “benefits”(Title/Abstract)OR “effectiveness”(Title/Abstract)OR “outcome”(Title/Abstract)OR “quality of life”(Title/Abstract))
**Filters**		Published up to February 22, 2026. No filters were applied.

The literature search was conducted without a start-date restriction, and all eligible studies published up to 22 February 2026, were included.

### Eligibility criteria

The inclusion criteria were chosen to identify journal articles on the effects, benefits, cost-effectiveness and overall impact of Namaste Care interventions on individuals with moderate to advanced dementia, medical personnel and family caregivers in any care setting (table 2). Studies evaluating family members, caregivers, healthcare staff or volunteers treating individuals with dementia who participate in Namaste Care were also included. Studies included adult participants with moderate to advanced dementia residing in any care setting with Namaste Care.

**Table 2:** Inclusion and exclusion criteria adopted during the systematic review search

Concept	Inclusion criteria	Exclusion criteria
Identification	Studies identifying or using economic tools (eg, cost-effectiveness analysis, cost-benefit analysis) and clinical assessment tools (eg, dementia severity scales, quality of life measures) to evaluate the outcomes of Namaste Care interventions.	Studies that do not include or explore the use of economic or clinical assessment tools to evaluate the outcomes of Namaste Care intervention.
Population	Individuals with moderate to advanced dementia, family members, caregivers, healthcare staff and volunteers involved in Namaste Care interventions.	Studies focusing on populations not participating in Namaste Care interventions with dementia.
Temporal scope	Studies published up to 22 February 2026.	Studies published after 22 February 2026.
Methodology	Peer-reviewed journal articles, systematic syntheses, (cluster) randomised controlled trials, qualitative studies, qualitative descriptive studies, methodologies, feasibility studies, overviews and small-scale studies.	Studies lacking primary data. Excludes review articles, meta-reviews, meta-synthesis, systematic reviews, narrative reviews, meta-analysis, protocols, commentaries, debates and editorials.
Intervention criteria	Studies addressing Namaste Care interventions implemented in nursing homes, care homes or home settings.	Studies not focusing on Namaste Care intervention in select settings.
Language	Published in English.	Non-English studies.

We included peer-reviewed journal articles, systematic syntheses, (cluster) randomised controlled trials, qualitative studies, qualitative descriptive studies, methodologies, feasibility studies, overviews and small-scale studies written in English. This systematic review excluded review articles, reviews, meta-reviews, systematic reviews and protocols, as well as studies without explicit mention of Namaste Care. It also excluded commentaries, debates and editorials.

### Study selection

**Figure 1:**
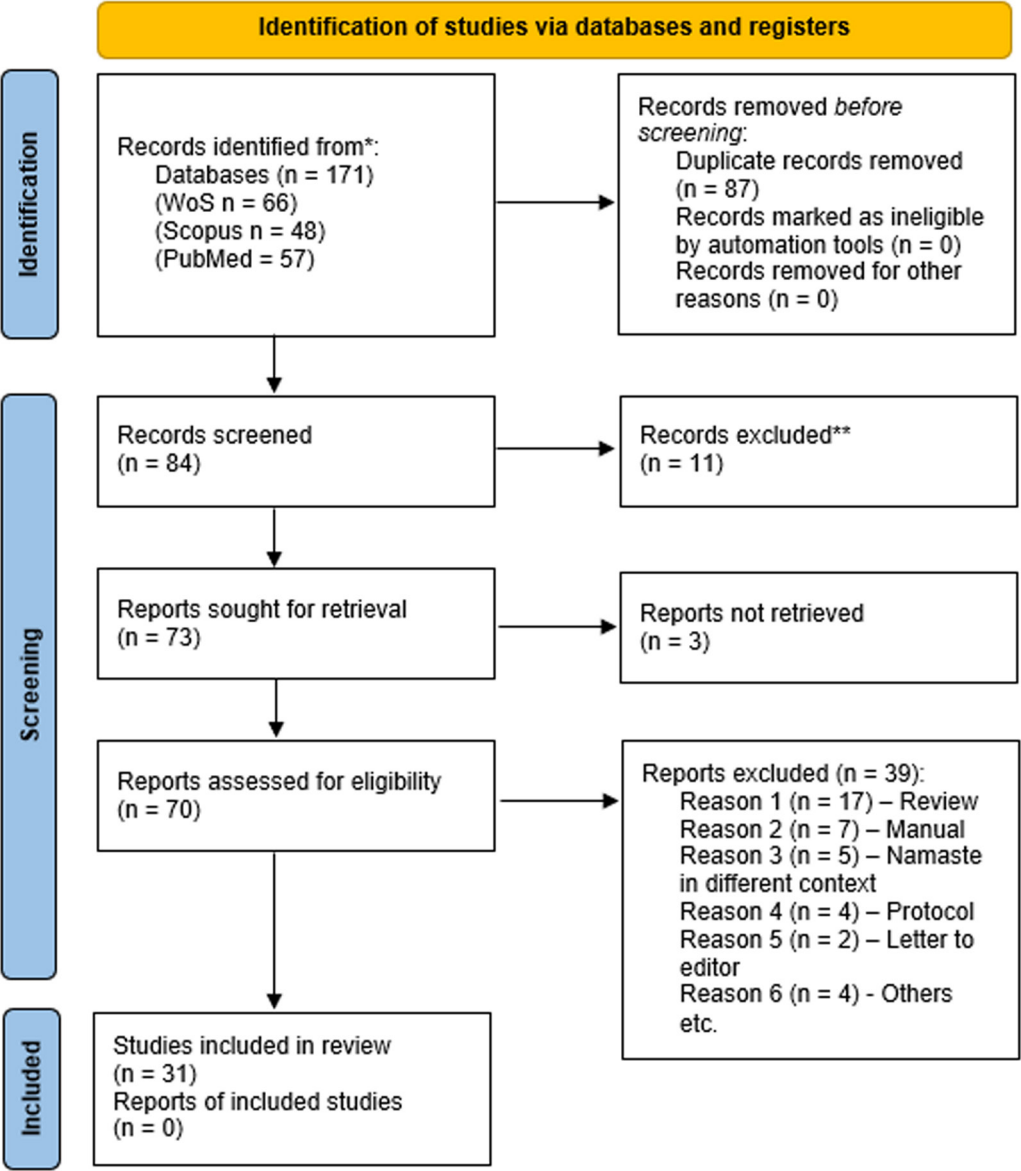
Preferred Reporting Items for Systematic Reviews and Meta-Analyses (PRISMA) flow diagram illustrating the study selection process for the systematic review.

Our initial database search identified 171 records. After removing 87 duplicate records, 84 records remained for screening. Titles and abstracts were screened independently by two reviewers (TP and JS). 11 records were excluded at this stage because they addressed topics unrelated to the review’s objectives. A total of 73 reports were sought for retrieval. Three reports could not be retrieved because full texts were not accessible. The remaining 70 full-text articles were independently assessed for eligibility by three reviewers (TP, JS and DT). Disagreements were resolved through discussion, and when necessary, a fourth reviewer (JH) was consulted. 39 full-text articles were excluded for predefined reasons. A total of 31 studies met the inclusion criteria and were included in the final review. Figure 1 illustrates the study selection process.

### Data extraction

A Microsoft Excel spreadsheet was designed and used to collect and categorise the study characteristics for this review. The following data were extracted from the studies: title, authors, abstract, type of research, publication year, aim/goal, location, number of healthcare facilities, type of respondents (residents, family, staff), sample size, resident sample size, staff sample size, family sample size, costs, benefits, clinical outcomes, QoL measure used, pain, neuropsychiatric symptoms, economic effects/outcomes, other benefits, intervention (specification), period intervention, evaluation methods, type of questionnaire and a uniform resource locator.

At least four members of the six members of the review team (TP, JS, DT, JH, LV and HJAS) independently assessed all the extracted data in all the studies to ensure thoroughness and accuracy. Any disagreements or discrepancies in the extracted data were resolved through discussion between at least two review team members to reach a consensus.

### Quality assessment

To assess the methodological quality of the included studies, we used the Mixed Methods Appraisal Tool (MMAT)^18^ for quantitative, qualitative and mixed-methods studies. For economic evaluation studies, the Consolidated Health Economic Evaluation Reporting Standards 2022 (CHEERS) were used to assess the completeness of reporting.^19^ Each study was independently evaluated by a team of three reviewers (TP, JS and DT). All disagreements in ratings were resolved by consensus. The results of the MMAT are presented in online supplemental data section 1, and the results of the CHEERS assessment are presented in online supplemental data section 2.

## Results

### Study characteristics

Online supplemental table 3 presents the characteristics of the 31 studies included. The studies are classified by various research methodologies, geographical locations, periods, data collection methods and sampling details.

#### Types of research design

The types of research designs varied considerably (online supplemental table 3). 8 studies employed a mixed-methods design, 15 were qualitative and 8 were quantitative. Four studies were trials, including three cluster randomised controlled trials (CRCTs). A pre/post design was frequently used (n=9) in quantitative and mixed-methods studies. Interviews were the most common data collection method across qualitative and mixed-methods studies (n=17), with semi-structured interviews being the predominant format (n=7). Focus groups were also frequently employed (n=7).

Five studies encompass economic evaluations, which also account for costs. Two studies address cost-effectiveness^5 20^; one study developed a cost model,^21^ which was also applied in another study by Bray *et al*.^20^

#### Geographical distribution

While the application of the Namaste Care programme is growing globally, the majority of studies originated from Europe and North America. A total of 10 studies originated from the UK, with one being a collaborative effort with the Netherlands.^810 11 20^,^26^ Nine studies are distributed in Canada^612^,^14 27^; seven of them are from Ontario.^6 12 14 27 28 30 31^ Six studies were conducted in the Netherlands.^25 11 32^,^34^ Other regions represented included the USA,^35 36^ Iran^9^ and Australia,^37 38^ two being from Tasmania,^39 40^ illustrating the increasing geographical reach of Namaste Care.

#### Variability in the duration of data collection

The studies show significant variability in data collection duration. For instance, the study by Karacsony *et al*^39^ had the shortest data collection period, lasting 3 days, focusing on staff education and training outcomes in Namaste Care. In contrast, studies by El Alili *et al*^5^ extended their data collection to 12 months to fully examine the economic impact on nursing home residents with dementia and their caregivers. Another study by Haaksma *et al*^11^ lasted 16 months. Overall, most studies had a data collection period of less than 1 year, with only a few extending beyond this timeframe. This variability reflects the diverse research methodologies and objectives of Namaste Care intervention studies.

#### Variability in sample size

The sample size variability is also evident, encompassing the number of nursing homes and the participants, including staff, family members and residents (online supplemental table 3). 15 studies were conducted in fewer than 5 nursing homes, and only 5 studies involved 10 or more nursing homes. Most often, research was carried out in two nursing homes, as seen in eight studies.^613 14 27^,^29 32 40^

The largest participant samples were reported by El Alili *et al*^5^ and Smaling *et al*,^34^ both with 231 residents and family caregivers. In contrast, the study by Bray *et al*^21^ was based on a much smaller sample of just eight residents.

The most extensive samples of nursing home staff included the studies by Froggatt *et al*,^24^ with 97 participants; Yous *et al*,^13^ with 58 participants; and Chang *et al*,^37^ with 49 participants. In contrast, McNiel and Westphal^35^ had a smaller sample size, with only 14 participants, while St John and Koffman^8^ had only 8 and Yous *et al*^31^ had 6 participants.

Studies focusing on family members also vary significantly. Studies by Li *et al*^6^ and Yous *et al*^14^ each included 42 family members, whereas de Vocht *et al*^32^ had a sample of four family members, and Bray *et al*^21^ had the smallest sample size of only one family member.

### Outcomes and effects of the Namaste Care intervention on residents

Our analysis categorised outcome measures into three distinct categories. The first category encompasses clinical outcomes, effects and benefits derived from quantitative studies and CRCTs, particularly those related to residents and caregivers. The second category focuses on economic costs, addressing the financial implications and costs associated with the interventions studied. Finally, the third category includes other benefits, drawing on insights from qualitative and mixed-methods studies. This categorisation provides a comprehensive framework for analysing and interpreting the diverse impacts characterised in the literature.

The included studies reported outcomes covering QoL, physical health, functionality, pain, mental health and overall well-being. Various outcome measurement instruments and methods have been employed to measure these outcomes. Online supplemental table 4 summarises the outcome measurement instruments used, as well as the observed economic impact, clinical effects, costs, benefits and other effects.

#### Quality of life and quality of dying

11 studies evaluated QoL and quality of dying (QoD) via 9 different outcome measurement instruments: the Quality of Life in Late-Stage Dementia (QUALID), Quality of Life for People with Dementia (QUALIDEM), EQ-5D-3L and EQ-5D-5L instruments, ICEpop CAPability Measure for Older People (ICECAP-O), ICECAP Supportive Care Measure (ICECAP-SCM), Carers-DEMentia Quality of Life (C-DEMQOL), End-of-Life care in Dementia-Comfort Assessment in Dying (EOLD-CAD) and the End-of-Life in Dementia-Satisfaction with Care (EOLD-SWC) (online supplemental table 4). The QUALID was the most common measure used in nine studies. EQ-5D-3L was used by El Alili *et al*.^5^ EQ-5D-5L, ICECAP-O and ICECAP-SCM were each used in one study by Froggatt *et al*.^24^ QUALIDEM^32^ and C-DEMQOL^12^ were also used once.

With respect to QoL effects, six studies reported no statistically significant benefits. In contrast, five studies demonstrated a statistically significant effect on the QoL of residents^9 23 24 40^ and caregivers^12^ (online supplemental table 4).

The QoL effects reported via the QUALID were mixed. Five studies^5 14 28 30 34^ reported statistically insignificant results, whereas three studies reported statistically significant positive effects.^23 24 40^ The effect on QoL was not statistically significant in a study that used the QUALIDEM.^32^ However, the results of a study using the C-DEMQOL were positive and statistically significant for both Caregiver Well-Being and Caregiver Role.^12^

Additionally, Froggatt *et al*^24^ used scales to assess EOLD. Specifically, the EOLD-CAD and EOLD-SWC scales were used. The results indicate that bereaved informal caregivers reported an improvement on the EOLD-CAD scale score 4 weeks after the patient’s death. These results are summarised in online supplemental table 4.

#### Clinical outcomes

Clinical and behavioural outcomes (excluding QoL) were investigated in 14 studies using a variety of outcome measurement instruments. Among these tools, some were used more frequently than others. For example, the Bedford Alzheimer Nursing Severity Scale was employed in four studies.^5 11 25 34^ The Charlson Comorbidity Index was used in three studies.^9 25 28^ The Minimum Data Set (MDS), among other tools,^36^ such as the Global Deterioration Scale,^23^ the Functional Assessment Staging Test,^24^ Behavioural codebook,^32^ The Older Persons and Informal Caregivers Survey Minimum Data Set (TOPICS-MDS),^5^ Visual Analogue Scale-Self-Rated Burden (SRB),^11^ Discomfort Scale - Dementia of the Alzheimer’s Type,^34^ SRB^34^ and Palliative Performance Scale^28^ were each used only once. The study by Karacsony *et al*^39^ was the only one that used Sense of Competence in Dementia Care Staff, Questionnaire on Palliative Care for Advanced Dementia and Palliative Approach for Nursing Assistants. For more details, see online supplemental table 4.

Pain was measured in five studies^14 24 25 28 30^; Pain Assessment in Advanced Dementia (PAIN-AD), Pain Assessment Checklist for Seniors with Limited Ability to Communicate-II (PACSLAC-II) and Doloplus-2 were used. A study by Froggatt *et al*^24^ indicated that the mean values of PAIN-AD decreased after 4 weeks in the Namaste Care group, although no statistical comparisons were carried out (this was a feasibility study). Studies by Kaasalainen *et al*^28^ and Yous *et al*,^14^ which used the PACSLAC-II, reported a reduction in pain, but the difference was not statistically significant. In contrast, a study by Stacpoole *et al*^25^ demonstrated a statistically significant correlation between pain scores measured with the Doloplus-2 and the severity of neuropsychiatric symptoms across all assessment points.

Similarly, tools for assessing behavioural symptoms were used in nine studies. The questionnaire for the neuropsychiatric inventory (NPI-Q) in Froggatt *et al*^24^ and Smaling *et al*^34^; the questionnaire for the Neuropsychiatric Inventory - Nursing Home Version (NPI-NH) in Froggatt *et al*^24^; Stacpoole *et al*^25^; Yous *et al*^14^ and Yous *et al*.^30^ In addition, the Cohen-Mansfield Agitation Inventory (CMAI) was used by Froggatt *et al*,^24^ Karacsony *et al*^40^ and Latham *et al*.^23^

The effects in the behavioural domain vary across studies. Only one study by Latham *et al*^23^ revealed statistically significant positive effects in the area of agitation, as measured by the CMAI, and increased positive well-being, as measured via the Namaste Short Questionnaire (NSQ). Other studies^14 24 25^ reported reductions in challenging behaviour, but the differences were not statistically significant.

The results of the NPI-NH and NPI-Q were not statistically significant in any study. The results for the CMAI were statistically significant in Latham *et al*^23^ and Karacsony *et al*^40^ but insignificant in Froggatt *et al*.^24^

#### Other outcomes

The Medication Quantification Scale was used in a study by Kaasalainen *et al*.^28^ The results reported were statistically significant and revealed a decrease in antidepressant use, especially benzodiazepine use. Additionally, the NSQ was employed by Bray *et al*^22^ to measure residents’ emotional well-being, physical well-being and awareness (reported by staff), who noted an improvement in the total NSQ score but did not test whether the improvement was statistically significant. The NSQ has also been used in studies by Latham *et al*^23^ and El Alili *et al*.^5^

### Outcomes and effects of the Namaste Care intervention on caregivers

We were also interested in other tools used in the studies aimed at caregivers, including the Global Appraisal of Individual Needs (GAIN), the Positive Aspects of Caregiving Scale (PAC), the Relational, Instrumental, Self-Soothing (RIS) Self-Efficacy Scale, the Zarit Burden Interview (with differing amounts of questions—ZBI-7 or ZBI-12), the Family Perception of Caregiving Role (FPCR) and the Family Visit Scale for Dementia (FAVS-D), which are also important for economic evaluation but are not widely incorporated into economic evaluations, which usually focus on patient outcomes.

Some of these studies focused on the burden of family caregivers. A study by Yous *et al*^14^ measured family carer role stress (eg, burden, guilt, conflict and loss) via the FPCR tool and quality of family visits between persons with dementia and family carers (FAVS-D). The study by Yous *et al*^14^ reported statistically significant improvements in both the FPCR and FAVS-D results. Additionally, Smaling *et al*^34^ found a significant positive effect of FPCR at 12 months.

Yous *et al*^12^ measured perceptions of caregiving via the 9-item PAC scale and used the Self-Efficacy Scales RIS-SE (Relational, Instrumental, Self-Soothing Eldercare Self-Efficacy scale) and ZBI-12 (Short-form Zarit Burden Interview scale). The results were statistically significant for all three measures.^12^ Interestingly, the results revealed higher ZBI-12 scores and caregiver burden for female caregivers than for male caregivers.

### Economic costs associated with Namaste Care

Five studies^5 20 21 24 28^ addressed the economic assessment of Namaste Care. Two studies compared the additional costs of Namaste Care to additional benefits in full economic evaluations.^5 20^ The remaining studies were partial economic evaluations focused only on the costs of implementation.^21 24 28^

Regarding the range of costs included, El Alili *et al*^5^ used a societal perspective wherein secondary care costs, medication costs, family costs and Namaste Care Family programme costs were identified, measured and valued. In contrast, Bray *et al*^20^ focused on programme costs only, using the resident level and session as the unit of analysis. Similarly, Froggatt *et al*^24^ focused on the healthcare resources used, valuing costs associated with primary care, secondary care, medication and informal care. Kaasalainen *et al*^28^ only evaluated the impact of Namaste Care on medication use and associated costs. In addition to cost analysis, Froggatt *et al*^24^ also identified the resource use elements that significantly contribute to the total cost of care per patient provided under each of the assessed options and used correlations to explore the strength of the relationship between economic measures and non-economic outcomes.

Across the studies, different time horizons were adopted when measuring costs post-intervention, with follow-up periods varying between 1–12 months. For example, El Alili *et al*^5^ reported costs at several points in time (1, 3, 6 and 12 months after baseline). Similarly, Froggatt *et al*^24^ reported costs at 4 weeks and 24 weeks post-intervention or following death, and Kaasalainen *et al*^28^ reported post-intervention effects at 3 and 5 months, respectively. Moreover, Bray *et al*^20 21^ reported costs as the mean incremental costs per session or per resident.

In full economic evaluations, El Alili *et al*^5^ conducted both cost-utility and cost-effectiveness analyses, comparing Namaste Care to usual care. EQ-5D scores were used to estimate quality-adjusted life years for the cost-utility analysis, while the cost-effectiveness analysis was based on QUALID and GAIN. By contrast, Bray *et al*^20^ focused on the costs of delivering the Namaste Care intervention. Using a theoretical cost model by Bray *et al*,^21^ they estimated that Namaste Care Intervention UK costs approximately £8–£10 more per resident per 2-hour session than a comparable period of usual care. This reflects higher intervention delivery costs rather than differences in broader healthcare resource use. In their cost-effectiveness analysis, higher implementation costs were, in some cases, associated with improvements in residents’ emotional well-being, physical well-being and awareness as measured by the NSQ.

Across cost analyses more broadly, Namaste Care was often associated with increased intervention-related costs in various categories,^20 21 24^ whereas Kaasalainen *et al*^28^ reported reductions in medication expenses, suggesting that potential cost offsets may arise primarily through changes in pharmacological management rather than through reductions in overall healthcare utilisation. Froggatt *et al*^24^ emphasised that future economic evaluations should prioritise the collection of data on key cost drivers, including general practitioner visits, inpatient stays, outpatient visits, ambulance services and medication use. They also reported that collecting resource-use data was feasible, although some concerns regarding data quality were noted.

### Qualitative benefits

A review of qualitative benefits was drawn from 28 studies (online supplemental table 4), comprising of 15 qualitative studies, 8 studies with mixed research designs and 5 quantitative studies that also reported observational or descriptive benefits. These benefits were primarily derived from semi-structured or in-depth interviews, focus groups, surveys with open-ended components and observational logs. Across studies, qualitative benefits were consistently reported for residents, family caregivers, staff and volunteers.

#### Residents

For residents, the most frequently reported qualitative benefits concerned improvements in emotional well-being and comfort, including increased calmness, relaxation and positive emotional responses such as smiling and enjoyment. Many studies also reported reduced agitation and increased responsiveness, along with greater engagement in meaningful and individualised activities. Improvements in social well-being were commonly reported, reflected in increased alertness, communication, responsiveness and interest in the environment. Several studies also reported perceived improvements in physical comfort and daily functioning, including better sleep, reduced pain and improved eating and drinking patterns (online supplemental table 4).

#### Volunteers and staff

Qualitative benefits for staff and volunteers primarily include increased job satisfaction, a sense of purpose and motivation to engage with Namaste Care. Studies described enhanced skills, confidence and awareness in caring for people with advanced dementia, as well as strengthened relationships with residents. Improvements in the working environment were frequently reported, including more opportunities for meaningful one-to-one time and a stronger focus on person-centred and comfort-oriented care (online supplemental table 4).

#### Family

Family caregivers consistently valued the personalised activities and emotional engagement associated with Namaste Care. Reported benefits included improved visit quality, greater reassurance about the quality and comfort of care and an enhanced emotional connection with their relatives. Many family members also described increased involvement in care and, in some cases, personal respite (online supplemental table 4).

## Discussion

Namaste Care is an emerging psychosocial intervention that is often used in nursing homes to improve the QoL of residents with dementia. This paper presents a systematic review to understand the clinical and economic effects, qualitative benefits and costs of Namaste Care in multiple care settings and to identify instruments used to measure these impacts. On the basis of a review of 31 eligible studies, we provided a comprehensive overview of the evidence to date. We show that the evidence concerning the clinical, economic and other costs and benefits is wide-ranging but leaves room for future scholarship to validate and extend the findings of existing scholarship. The geographical distribution of Namaste Care studies in our analysis is notably wide, with studies emerging from regions as diverse as Europe, North America, Australia and the Middle East. Our review was restricted to English-language studies to ensure consistency and accuracy in data extraction. This restriction was expected to have minimal impact on the overall findings. Although we identified one study published in Persian, it was not included in the systematic review due to language limitations. A further limitation concerns the inclusion of studies regardless of methodological quality. This was a deliberate protocol-specified decision to ensure comprehensive mapping of the evidence. In our assessment (MMAT, online supplemental data section 1), we indicate that only three studies^21 36 40^ met approximately 60% of the applicable MMAT criteria, while all remaining studies met 80% or 100% of the criteria, which has a very limited impact on the overall findings of our study. To address the potential impact of this approach on study inferences, we conducted a sensitivity analysis restricted to higher-quality studies—primarily the three randomised controlled trials: Froggatt *et al*,^24^ El Alili *et al*,^5^ Smaling *et al*^34^ and the stronger mixed-methods studies. The core directional conclusions of the review remained unchanged: statistically significant QoL benefits were limited, cost data indicated modest increases in resource use with potential medication savings and qualitative benefits to residents and caregivers were consistently reported. However, restricting to higher-quality studies substantially narrows the evidence base, as much of the literature derives from feasibility and pilot studies further underscoring the need for larger, methodologically rigorous trials.

Namaste Care has been evaluated via several different research methods, including mixed, quantitative and qualitative research methods. The majority of studies were observational, with just three CRCTs published to date. Studies have employed different outcome measurement instruments, including clinical, economic and other domains. Only 11 studies reported QoL and QoD outcomes, which were measured most commonly using the QUALID scale (9 out of 11). This suggests an emerging consensus about the appropriateness of this instrument, which has been designed specifically for people living with dementia. Research by Sopina *et al*^41^ has shown that the QUALID has distinct advantages over other QoL measures in the dementia population and has excellent psychometric properties.^42^ However, it is not preference-based (which limits its use in economic evaluations). Our review identified a range of outcome measures used by studies of Namaste Care, commonly including measures of pain, behavioural symptoms, dementia severity, comorbidity and other outcomes. These may be relevant to different types of analyses, ranging from cost-effectiveness studies to broader cost-consequence analyses and may inform reimbursement decisions by national authorities, particularly those that use multiple-criteria decision analysis to allocate limited resources in vital areas such as palliative care.

Different perspectives were used in these studies, including the societal perspective, for example, El Alili *et al*,^5^ and the healthcare resources perspective by Bray *et al*.^20^ These differing methodologies do not allow for the results to be compared directly, but the results expand the map of cost analysis and better inform the implementation of Namaste Care. Bray *et al*^21^ conducted a cost analysis for the resource consumption of the Namaste Care intervention, including staff costs, capital costs and consumable costs. Their full cost model indicated that Namaste Care intervention in the UK costs approximately £8–£10 more per resident per 2-hour session than does a comparable period of usual care.

Studies by El Alili *et al*,^5^ Simard and Volicer^36^ and Kaasalainen *et al*^28^ have frequently reported that medication costs contribute to cost differences, with indications that the prescription of pain medications and antidepressants may decrease as a result of Namaste Care. These findings suggest that the intervention may have a positive effect on both medication usage and the overall well-being of patients. Future work may expand on these findings in larger study samples in different countries and/or in different care settings.

The most comprehensive cost-effectiveness finding to date, drawing on a randomised control trial that included 231 residents, is provided by El Alili *et al*,^5^ which indicates no statistically significant differences in costs and clinical outcomes (eg, QoL and positive family caregiving experiences) between the Namaste Care Family programme and usual care. However, the authors noted that overall clinical outcomes may improve and costs may decrease due to the Namaste Care Family programme. The authors noted that when considering statistical uncertainty, the probability of the Namaste Care Family programme being cost-effective compared with usual care at a ceiling ratio of 0 € per additional unit of effect was 0.70.

The strengths of Namaste Care have been explored by numerous studies, with an increase in publications over time reflecting a growing interest in Namaste Care. However, the heterogeneity of measurement tools and methods and the variety of Namaste Care implementations make it difficult to evaluate the generalisability of their conclusions. Furthermore, it is evident that some aspects of Namaste Care have not been scrutinised. Research documents that implementing and sustaining the intervention is a challenge,^6^ with session duration and frequency often being cited as the driving culprit.^31 33^ Semi-structured interviews revealed that staff and family caregivers at times felt overwhelmed by Namaste Care sessions.^6^ Data collection and consistency are issues that affect research of vulnerable populations in settings with limited resources, and future work should be mindful of these challenges.^43 44^

Owing to the difficulties in implementing Namaste Care, alterations in the intervention occur to sustain it.^6^ Therefore, since Joyce Simard crafted the Namaste Care intervention to improve daily QoL and not as a programme that participants have access to once or two times a week, it would be useful to define when, in fact, Namaste Care is the intervention being delivered.^15^ Clear guidelines on the minimal requirements (eg, minimal and optimal doses^23^) of Namaste Care may help future research evaluate the intervention in a more consistent manner. Studies that fail to research daily interventions or significantly reduce the session duration, such as Li *et al*,^6^ present results that may not correctly reflect the impact of Namaste Care. This raises a critical question: whether the intervention being implemented under the label of Namaste Care in these studies truly adheres to the core principles and guidelines originally outlined by Simard.^4^ Knowing precisely what constitutes Namaste Care will not only lead to better comparisons but also make it possible to more clearly evaluate the cost-effectiveness of the intervention. The heterogeneity of outcomes, outcome measurement instruments and different research methodologies used in the studies reviewed present a barrier to generalising their results in any specific domain, such as QoL.

This review identified a range of outcome measurement instruments as well as economic, clinical and additional outcomes of Namaste Care in published studies. Future work may consider exploring the delivery of Namaste Care itself (including aspects related to its minimal and optimal doses) as well as reporting results via common outcome measurement instruments, which would increase comparability across studies of Namaste Care. In areas such as Alzheimer’s disease, regulatory bodies such as the US Food and Drug Administration publish guidance documents specifying the types of clinical endpoints that would be acceptable for safety and efficacy evaluation,^45^ which have an empirically confirmed impact on clinical trial design^46^; however, to our knowledge, no such guidelines have been published for palliative care interventions in Europe or the USA.

Future work should also consider generating more comprehensive evidence on the economic costs and benefits of Namaste Care as well as the feasibility of implementing it in different care settings. To date, few studies have conducted formal cost-effectiveness analyses of dementia palliative care interventions, as shown by O’Connor *et al*.^47^ This study identified seven studies that included economic outcomes via a range of methodologies, including cost-effectiveness analysis, cost modelling, partial economic evaluation and a feasibility study. Other analytical approaches, such as cost-consequence analysis, assess ‘a wide range of costs and consequences (effects) and report them separately. It includes all types of effects, including health, non-health, negative and positive effects, both to patients and other parties (for example, caregivers)’.^48^ These approaches may be considered by researchers in the future, given the complexity of an economic evaluation that draws on a range of outcomes, as this review showed was common for Namaste Care.

## Conclusion

Dementia and other neurodegenerative conditions present unique challenges in our ageing societies. The rising numbers of people with these conditions requiring nursing support and care have been a concern for many healthcare and social care systems. There is a growing need to understand the benefits of non-pharmaceutical interventions to improve the QoL and dignity of those living with these conditions as well as their caregivers. Namaste Care may be considered one such intervention.

In our systematic review, we catalogued the various tools used to measure the effects and benefits of the Namaste Care intervention. We have shown a high level of variation in the assessment tools used and the context, as well as how and where they are used.

Notably, few studies have evaluated Namaste Care using clinical outcomes that can be applied for economic assessments. Additionally, many of the reported outcomes lacked statistical significance, limiting their utility in establishing evidence-based conclusions. Given the unique nature of Namaste Care as a specialised intervention, assessing its economic impact presents inherent challenges. Therefore, it is essential to employ validated outcome measurement instruments that are appropriate for capturing both clinical and economic dimensions. A deeper understanding of these instruments and their applicability to Namaste Care is crucial for advancing research in this field and may be subject to further study.

Second, further work may consider the effects of Namaste Care on medication usage and could elucidate the mechanisms underlying the potential for reducing its utilisation and its implications for both patient outcomes and economic efficiency. By addressing these gaps, future studies can contribute to a more robust evidence base for the clinical and economic evaluation of Namaste Care, thereby supporting its integration into broader healthcare strategies.

Although some research findings suggest that Namaste Care may improve the QoL of those living with dementia in nursing homes, the variability in outcome measurement instruments and the different research methodologies used present a barrier to generalising their results. Understanding the optimal dose of the intervention as well as standardising the evaluation of the intervention by using comprehensive instruments in sufficiently powered sample sizes could lead to more comparable and, therefore, more reliable data in support of both clinical and economic evaluation of Namaste Care.

## Supplementary material

10.1136/bmjopen-2025-102899 online supplemental file 1

## Data Availability

All data relevant to the study are included in the article or uploaded as supplementary information.
