# Supplementary material for: Exploring the impact of Namaste Care for individuals with advanced dementia: a systematic review of costs, effects and benefits
Source: BMJ Open. 2026 May 6;16(5):e102899. doi: 10.1136/bmjopen-2025-102899 (PMC13150879; doi:10.1136/bmjopen-2025-102899)
Supplement: online supplemental file 1 [file bmjopen-16-5-s001.pdf]

SUPPLEMENTAL MATERIAL

Table 3: Characteristics of the Included Studies

Study	Design, method	Country, City/Region	Time Period	Duration of Data Collection	No. of Nursing Homes	Participants Residents/Staff/Family
Bray et al. [21]	Quantitative, cost model	The UK	Not mentioned	Not mentioned	1	8/0/1
Bray et al. [20]	Quantitative, cost model	The UK	Not mentioned	3 and 6 months	5	48/0/0
Bray et al. [22]	Qualitative descriptive, survey	The UK	2016	4 weeks	4	0/20 directly practiced, 23 observed in practice, 57 heard of NC but had not seen it practiced/0
Chang et al. [37]	Qualitative, focus groups	Australia, New South Wales	2011	6 months	8	40/49 (30 RN, 19 AN)/8
Dalkin et al. [10]	Qualitative, focus groups, Interviews	The UK, Northeast England	Not mentioned	Not mentioned	1*	X/0/8
De Vocht et al. [32]	Mixed, pre-/post-test, interviews	The Netherlands	August 15, 2014 - December 19, 2014	18 weeks	2	15/0/4
El Alili et al. [5]	Quantitative, cluster-randomized controlled trial	The Netherlands	May 2016 - December 2018	12 months	19 (NCF program n=10, usual care n=9)	231/0/231
Froggatt et al. [24]	Mixed, HTA, feasibility study, cluster-randomized controlled trial, interviews	The UK, Northwest England	August 2017 - November 2017	24 weeks	8	32/ 97/12 (friend or family)
Haaksma et al. [11]	Qualitative, interviews	The UK (London area), and The Netherlands	Preparation Phase: Dec 2017 - Oct 2018, Pilot Phase: Sep 2018 - Mar 2019	16 months	10	Preparation phase: 0/17 nursing staff, 3 program coordinators, 6 nursing home managers, 2 volunteers/8<br>Pilot phase: 1/13 volunteers/12
Kaasalainen et al. [27]	Qualitative, survey, interviews	Canada, Ontario and Saskatchewan	Funds granted between 2015-2017	3 months	2	0/44 survey respondents, 18 interviewees/44 survey respondents, 5 interviewees
Kaasalainen et al. [28]	Mixed, pre-/post-test study, interviews	Canada, Ontario and Saskatchewan	18 months	6 months	2	31/28, 6 volunteers/10
Karacsony et al. [39]	Mixed, pre-/post-test, survey, semi-structured interviews and focus groups	Northern Tasmania	Not mentioned	3 days	1	0/35/0

Karacsony et al. [40]	Mixed-methods intervention study; four-stage implementation design; quantitative repeated measures (QUALID, CMAI) + qualitative observational logs; linear mixed effects models	Australia, Tasmania	Residents enrolled gradually over a six-month period; intervention evaluated at baseline, 2, 4 and 12 weeks	12-week intervention (with recruitment occurring over 6 months)	2	Recruited 10, completed 7/0/0
Kohne et al. [9]	Quantitative, clinical trial	Iran, Tohid, Tehran	2017	6 months	1	25/0/0
Latham et al. [23]	Mixed, pre-/post-test, semi-structured interviews	The UK	February 2017 - April 2019	12, 18, or 24 weeks	6 (n=5 completed)	Recruited 48, included in final data set 36/15 questionnaires, 29 reflective diaries, 19 interviews/9
Li et al. [6]	Qualitative, semi-structured interviews	Canada, Southern Ontario	November 2017 - April 2018	5 months	2	53/46/42
McNiel and Westphal [35]	Qualitative, interviews	USA	Not mentioned	12 months	Not mentioned	0/14/0
Nicholls et al. [38]	Qualitative, focus groups	Australia, Metropolitan and regional areas of NSW	Not mentioned	6 months	3 RACFs	0/0/11
Simard and Volicer [36]	Quantitative study with qualitative/observational elements	USA, Massachusetts	Not mentioned	6 months	6	86/0/0
Smaling et al. [2]	Qualitative, semi-structured interviews	The Netherlands	December 2017 - October 2018	12 months, 2 homes dropped out after 3 and 6 months	10	0/31/12
Smaling et al. [34]	Quantitative, cluster-randomized controlled trial	The Netherlands	December 2016 - December 2018	1, 3, 6, and 12 months follow-up	19	231/0/231
St John and Koffman [8]	Qualitative, individual, semi-structured and face-to-face interviews	The UK	Not mentioned	2 weeks	5	0/8/0
Stacpoole et al. [25]	Quantitative non-randomized study, action research	The UK, London	February 2012	4-6 months	6	37 (30 surviving)/0/0
Stacpoole et al. [26]	Qualitative, focus groups and interviews, pre-post design	The UK, London	Not mentioned, recruitment started in 2012	Not mentioned	6	37/0/0

Tasseron-Dries et al. [33]	Qualitative, semi-structured interviews	The Netherlands	December 2017 - October 2018	10 months	10	10/31 staff, 2 volunteers/10 family caregivers
Yous et al. [12]	Multiphase mixed	Canada, Ontario	October 2020 - September 2021	3 months	Not applicable	12/0/12
Yous et al. [13]	Qualitative, focus groups and interviews	Canada	October 2017 - April 2018	6 months	2	X/58/10
Yous et al. [14]	Quantitative, pre-/post-test	Canada, Ontario	Not mentioned	After 3 and after 6 months	2	53/0/42
Yous et al. [29]	Qualitative, semi-structured interviews	Canada	Not mentioned	6 months	2	53/21 (5 staff, 4 administrators, 12 volunteers)/16
Yous et al. [30]	Mixed methods multiphase design, focus group, interviews, pre-/post-test	Canada, Ontario	June 2018 - April 2019	6 months: baseline, 3 months, and 6 months	1 hospital-based specialized dementia care unit	15/22 staff, 4 volunteers/14
Yous et al. [31]	Qualitative descriptive study; participatory adaptation workshops; thematic analysis	Canada, Ontario	October 2020 - January 2021	two sessions per caregiver, each 60–90 min	Not applicable	7/0/6

*Note: X - Not mentioned, * - A hospice in Northeast England has made provisions to provide NC in the person's own home, RN - Registered Nurses, AC - Assistants in Nursing, NC - Namaste Care*

Table 4 Outcome Measurement Instruments Used, Reported Outcomes, Observed Effects, Costs, and Benefits

Study	OMIs used	Economic effects/outcomes/costs	Observed clinical and other effects	Benefits
Bray et al. [21]	Not included	• using a cost model, Namaste Care (NC) sessions were valued at £8-£10 more costly per resident per 2-hour session compared to usual care<br>• providing NC will also have other associated costs/benefits compensating for the additional cost of the intervention	Not included	Not included
Bray et al. [20]	*Other OMIs*<br>• NSQ	• NC additional costs: £7.24 per resident/session<br>• if outcomes ↑ with usual care:<br>– extra £4.46 per resident offered no NSQ score benefit<br>• if usual care had no impact:<br>– extra £5.53 per resident ↑ NSQ scores by 1 point<br>• if outcomes declined with usual care:<br>– extra £1.22 per resident ↑ NSQ scores by 1 point	• NSQ: average improvement of 1.31 points for the total NSQ score	*Residents:*<br>• ↑ physical well-being<br>• ↑ social well-being<br>• ↑ emotional well-being
Bray et al. [22]	Not included	Not included	Not included	*Residents:*<br>• ↑ emotional well-being (calmness, relaxation, ↑ mood, ↑ smiling)<br>• ↓ agitation and pain<br>• ↑ physical health and comfort (skin condition, hydration, sleep, swallowing)<br>• ↑ social interaction, responsiveness, and communication<br>*Staff:*<br>• ↑ job satisfaction and positive attitudes<br>• ↑ person-centered and relational care culture<br>*Family carers:*<br>• ↑ comfort and engagement of family members

Chang et al. [37]	Not included	Not included	Not included	*Residents:*<br>• ↓ falls and minor injuries<br>• ↑ calmness and settled behavior<br>• ↑ vocalization, activity, and engagement<br>• ↑ eating and sleeping patterns<br>*Staff:*<br>• ↓ time spent on wound care
Dalkin et al. [10]	Not included	Not included	Not included	*Residents:*<br>• ↑ emotional connection and attachment<br>• ↑ familiarity with surroundings and carers<br>• ↑ engagement and ↓ social isolation<br>• ↑ individualized and tailored care<br>*Volunteers:*<br>• ↑ meaningful relationships with people with dementia<br>• ↑ sense of purpose and satisfaction<br>*Family carers:*<br>• ↑ personal respite<br>• ↑ satisfaction with personalized activities and emotional engagement
De Vocht et al. [32]	*OMIs of QoL and QoD*<br>• QUALIDEM<br>*OMIs for clinical and behavioral outcomes*<br>• Behavioral codebook	Not included	• QUALIDEM: no significant differences in the dimensions of quality of life during the study period	*Residents:*<br>• ↑ positive behavior and responsiveness (i.e. eye contact, touch, verbal response, tracking stimuli) → ↑ QoL<br>• ↓ fear and social isolation
El Alili et al. [5]	*OMIs of QoL and QoD*<br>• QUALID<br>• EQ-5D-3L<br>*OMIs for clinical outcomes*<br>• BANS-S<br>*Other OMIs*<br>• GAIN<br>• NSQ<br>• TOPICS-MDS	• NCF program is more cost-effective than usual care<br>• most significant economic effect: medication reduction (−1,002 €, 95% CI: −3,971 to 1,268)<br>• total healthcare costs lower in NCF, but not significant (−1,111 €, 95% CI: −4,071 to 1,246)<br>• mean intervention costs: 793 € per NCF participant<br>• 1 point improvement in GAIN score cost savings of 7,310 €	• QUALID: no significant differences<br>– $\bar{d}$ = -0.060 (-0.42; 0.30)<br>• EQ-5D-3L: no significant differences<br>– $\bar{d}$ = 0.015 (-0.058; 0.088)<br>• BANS-S: $\bar{x}$ = 14.7 (4.8)<br>• GAIN: no significant differences,<br>– $\bar{d}$ = 0.033 (-0.24; 0.31)	Not included

		• 1 point improvement in QUALID saves 8,919 €<br>• cost-utility analysis: each gained QoL saves 315,671 €		
Froggatt et al. [24]	*OMIs of QoL and QoD*<br>• QUALID<br>• EQ-5D-5L<br>• ICECAP-O<br>• ICECAP-SCM<br>• EOLD-CAD<br>• EOLD-SWC<br>*OMIs for clinical and behavioral outcomes*<br>• FAST<br>• PAIN-AD<br>• NPI-Q<br>• CMAI<br>• NPI-NH<br>• PACSLAC-II<br>*Others*<br>• GAIN	• 2 homes had no additional costs, 2 others ↑ spending by £222 per resident over 4 weeks<br>• QUALID had a stronger correlation with economic outcomes than CMAI; particularly with ICECAP-SCM<br>• clear correlation pattern for economic measures with CMAI<br>• costs for NC included: primary care, secondary care, medication, informal care<br>• main cost drivers: GP visits, inpatient stays, outpatient visits, ambulance costs, and medication	• QUALID: ↓ x̄ slightly after 4 weeks in both groups; at 24 weeks, ↓ continues the NC group, and ↑ again in the control group<br>• EOLD-CAD: ↑ x̄ slightly after 4 weeks and then decreases but remains higher than at baseline at 24 weeks<br>• EOLD-SWC: ↓ x̄ slightly overtime<br>• NPI-Q, CMAI, PAIN-AD: ↓ observed mean values after 4 weeks in the NC group, although no statistical comparisons were carried out<br>• EQ-5D-5L, ICECAP-O, ICECAP-SCM: used for economic evaluation	*Residents:*<br>• ↑ QoL (greater awareness and engagement with family, staff, and surroundings)<br>• ↑ relaxation<br>• ↓ agitation<br>*Staff and informal carers:*<br>• ↑ quality time with residents<br>• ↑ quality of care through one-to-one time
Haaksma et al. [11]	*OMIs for clinical outcomes*<br>• BANS-S<br>• VAS-SRB	Not included	• BANS-S: x̄ = 15 (5)<br>• VAS: x̄ = 7 (2)	*Residents:*<br>• ↑ mood and social interaction<br>• ↓ pain after a massage<br>• ↑ engagement and independence<br>• ↑ liquid intake<br>*Family carers:*<br>• ↑ enjoyment and relaxation during NC sessions<br>• ↑ calm presence and physical contact
Kaasalainen et al. [27]	Not included	Not included	Not included	*Residents:*<br>• ↑ mood, engagement, and alertness<br>*Staff and family carers:*<br>• ↑ engagement and empowerment

Kaasalainen et al. [28]	*OMIs of QoL and QoD*<br>• QUALID<br>*OMIs for clinical outcomes*<br>• CCI (Charlson comorbidity index)<br>• PACSLAC-II<br>• PPS<br>*Other OMIs*<br>• MQS	• medication costs ↓ slightly from baseline to post-intervention: $\bar{d}$ = $ - 5.8 (53.6)	• CCI: $\bar{x}$ = 4.3 (1.7)<br>• QUALID: no significant differences $\bar{d}$ = -1.7 (10.9)<br>• PACSLAC-II: no significant differences $\bar{d}$ = -0.7 (4.3)<br>• MQS: statistically significant ↓ in antidepressant use (t = 1.89, p = 0.05)<br>− total score $\bar{d}$ =-0.7 (11.4)<br>− Benzodiazepine $\bar{d}$ = -1.7 (10.9)<br>− Antidepressant $\bar{d}$ = -0.4 (3.3)<br>− Antipsychotic $\bar{d}$ = 0.0 (3.1)<br>− Acetaminophen $\bar{d}$ = 0.1 (2.7)<br>− NSAIDs $\bar{d}$ = -0.1 (2.7)	*Residents:*<br>• ↑ mood<br>• ↑ engagement<br>• ↑ alertness<br>*Staff and family carers:*<br>• ↑ engagement and empowerment<br>• ↑ mood<br>• ↑ positive moments and interactions with staff and family members
Karacsony et al. [39]	*OMIs for clinical outcomes*<br>• SCIDS<br>*Other OMIs*<br>• qPAD<br>• PANA	Not included	• SCIDS: statistically significant↑ in building relationships subscale<br>• qPAD:<br>− statistically significant ↑ in knowledge (p<0.01)<br>− statistically significant ↑ in attitudes towards palliative care (p<0.01)<br>• PANA: statistically significant ↑ in self-reported care skills (p < 0.05)	*Residents:*<br>• ↑ QoL (comfort, calmness, pleasure)<br>• ↓ pain and anxiety, and responsive behaviors (e.g. agitation, rejection of care)<br>• ↑ social engagement and meaningful interaction<br>*Family carers:*<br>• ↑ reassurance about the quality and comfort of care<br>• ↑ involvement in meaningful activities<br>*Staff:*<br>• ↑ knowledge and skills in advanced dementia and palliative care<br>• ↑ confidence and competence in person-centered care<br>• ↑ positive attitudes and relationship-building with residents
Karacsony et al. [40]	*OMIs of QoL and QoD*<br>• QUALID<br>*OMIs for clinical outcomes*<br>• CMAI	Not included	• QUALID: significant ↑ at week 2 and week 4 vs. baseline (p = 0.004; p = 0.009); borderline improvement at week 12 (p = 0.07)<br>• CMAI: significant ↓ in agitation at week 2 (p = 0.02) and week 12 (p = 0.01) vs baseline	*Residents:*<br>• ↑ calmness and comfort<br>• ↓ agitation and distress<br>• ↑ mood and positive emotional responses (e.g., smiling)<br>• ↑ engagement and social interaction<br>• ↑ enjoyment of sensory and social activities
Kohne et al. [9]	*OMIs of QoL and QoD*<br>• QUALID	Not included	• QUALID: significant ↓ in total QoL score<br>− 18.72 ± 1.01 at the end of trial compared with 25.64 ± 1.64, at baseline p<0.001	Not included

	OMIs for clinical outcomes<br>• CCI			
Latham et al. [23]	*OMIs of QoL and QoD*<br>• QUALID<br>*OMIs for clinical outcomes*<br>• GDS<br>• CMAI<br>*Other OMIs*<br>• NSQ	Not included	• QUALID: significant positive effects (t = 2.92, p = 0.01, n = 31) $\bar{d}$ = -4.29 (-0.12)<br>• SR = 5<br>• CMAI: significant positive effects (t = 3.31, p = 0.002, n = 32) $\bar{d}$ = -4.81 (-0.13)<br>• NSQ: ↑physical well-being, emotional well-being, alertness/awareness (Improve = 22. Stable = 14)<br>• medication: analgesia, sedatives and antipsychotics - few changes during intervention	*Residents:*<br>• ↑ physical and mental well-being<br>• ↑ responsiveness and connection<br>*Staff:*<br>• ↑ sense of purpose and well-being<br>• ↑ positive relationships<br>*Family carers:*<br>• ↑ perceived positive changes in their relative<br>• ↑ relationships and involvement<br>*Care home:*<br>• ↑ reputation<br>• ↑ culture of continuous improvement
Li et al. [6]	Not included	Not included	Not included	*Staff:*<br>• ↑ involvement in dementia care<br>• ↑ personal time with residents<br>• ↑ understanding of residents and their needs
McNiel and Westphal [35]	Not included	Not included	Not included	*Residents:*<br>• ↑ calmness, relaxation, and positive emotional responses (Peaceful Sanctuary effect)<br>*Staff:*<br>• ↑ job satisfaction<br>• ↑ reinforcement of nursing values
Nicholls et al. [38]	Not included	Not included	Not included	*Residents:*<br>• ↑ mental well-being through gentle touch<br>*Family and staff:*<br>• ↑ relaxation and comfort during interactions

Simard and Volicer [36]	*OMIs for clinical outcomes*<br>• MDS<br>• CPS (from MDS)<br>• DRS (from MDS)<br>• CBP (from MDS)<br>• Delirium Indicators (from MDS)	Not included	• MDS<br>– no significant differences in depression scores and between behavioral symptoms<br>– ↑ social interaction (3.27 + 0.30 vs 2.00 + 0.47, p = 0.046)<br>– ↓ total indicators of delirium after enrollment (2.52 + 1.94 vs 2.35 + 1.88, p = 0.079)<br>– no significant difference in the administration days for antipsychotics, antidepressants, or hypnotics<br>– significant ↓ in the administration days for antianxiety medications (0.80 + 2.18 vs 0.49 + 1.79, p = 0.035)<br>– slight ↓ in the number of residents receiving antidepressants	*Residents:*<br>• ↑ social interaction / interest in environment<br>• ↓ delirium indicators<br>• ↓ agitation (trend)<br>• ↓ antianxiety medication use<br>• ↑ quality of life / comfort<br>*Family:*<br>• ↑ visit quality<br>• ↑ meaningful engagement with relatives
Smaling et al. [2]	Not included	Not included	Not included	*Residents:*<br>• ↑ physical and emotional well-being, more engagement, enhanced interactions, changes in energy level, and weight gain<br>*Family caregivers:*<br>• more positive view of people with dementia, changes in family visits, mixed feelings during sessions, and mixed changes in relations with all others<br>*Staff:*<br>• diverse work experiences shift to more person-centered care (more time and attention for residents, and more awareness), and developing relationships with residents and colleagues
Smaling et al. [34]	*OMIs of QoL and QoD*<br>• QUALID<br>*OMIs for clinical and behavioral outcomes*<br>• BANS-S<br>• NPI-Q<br>• DS-DAT<br>*Others*<br>• ZBI-7<br>• FPCR	Not included	*OMIs of QoL and QoD*<br>• QUALID: no significant overall effect on residents' QoL, very small effect size (Cohen's d = 0.09)<br>*OMIs for clinical outcomes*<br>• BANS-S: descriptive only (no effect analysis)<br>• NPI-Q: no significant effect on challenging behavior; small effect size (Cohen's d = 0.15)<br>• DS-DAT: significant ↓ in discomfort (overall)	*Residents:*<br>• ↓ discomfort<br>• fewer distressing medical events (especially pneumonia)<br>• ↑ comfort and well-being<br>• more opportunities for meaningful and person-centered activities<br>*Family caregivers:*<br>• ↓ conflict with staff over caregiving

	• GAIN<br>• SRB		– significant positive effect (p = 0.012); Cohen's d = −0.22; stronger effect at 12 months: Cohen's d = −0.42<br>*Others*<br>• ZBI-7: no significant effect; Cohen's d = −0.12<br>• FPCR: significant positive effect at 12 months (p = 0.049); Cohen's d = −0.28<br>• GAIN: no significant effect; Cohen's d = −0.05<br>• SRB: no significant effect; Cohen's d = −0.12	• ↑ collaboration and communication with nursing staff<br>• reassurance about the quality and comfort of care<br>• facilitated meaningful connection with the person with dementia<br>*Staff:*<br>• ↑ awareness of residents' needs and distress<br>• ↑ focus on comfort-oriented and person-centered care<br>• *s*upport for a palliative care approach<br>• ↑ collaboration with family caregivers
St John and Koffman [8]	Not included	Not included	Not included	*Residents:*<br>• ↓ in agitated behavior and ↑ emotional well-being<br>• ↑ calmness and ↑ relaxation<br>• ↑ connection and communication via sensory interaction
Stacpoole et al. [25]	*OMIs for clinical and behavioral outcomes*<br>• BANS-S<br>• NPI-NH<br>• CCI<br>• Doloplus-2	Not included	• CCI and BANS-S: no correlation between BANS-S and/or CCI with NPI-NH scores<br>• NPI-NH: ↓ in 4 care homes but ↑ in 1 care home<br>• Doloplus-2: significant correlation between pain scores and symptom severity at all assessments (baseline r = 0.605, p<0.001; time interval 2 r = 0.793, p<0.001; time interval 3 r = 0.605, p = 0.001; time 4 r = 0.814, p<0.001)	*Residents:*<br>• ↓ behavioral symptoms (agitation, aggression, anxiety, irritability)<br>• ↓ pain<br>• ↑ comfort and calmness<br>• ↑ quality of life
Stacpoole et al. [26]	Not included	Not included	Not included	*Residents:*<br>• chaos and confusion were replaced with calmness<br>• rushing around was replaced with reaching out to each other<br>• lack of trust was replaced with seeing the person<br>• more person-centered, calm and supportive care environment enhancing overall well-being

Tasseron-Dries et al. [33]	Not included	Not included	Not included	• ↑ mood of residents, family, and staff<br>*Family:*<br>• created meaningful connections with their relatives and ↑ satisfaction<br>• ↑ spontaneous participation<br>• ↑ willingness to be involved
Yous et al. [12]	*OMIs of QoL and QoD*<br>• C-DEMQOL<br>*Other OMIs*<br>• PAC<br>• RIS-SE<br>• ZBI-12	Not included	Significant effects for all<br>• C-DEMQOL Career Well-being: $\bar{d}$ = -1.10 (-2.47, 0.27)<br>• C-DEMQOL Career Role: $\bar{d}$ = -0.20 (-1.14, 0.74)<br>• PAC: $\bar{d}$ = 1.20 (-3.36, 5.76)<br>• RIS-SE $\bar{d}$ = 0.70 (-1.98, 3.38)<br>• ZBI-12: $\bar{d}$ = -0.50 (-4.26, 3.26)	*Residents:*<br>• ↑ engagement in meaningful activities consistently<br>• ↑ well-being<br>• the NC program instilled confidence in people living with moderate dementia to participate in different activities
Yous et al. [13]	Not included	Not included	Not included	*Residents:*<br>• a designated quiet space and a small group format were helpful for engagement
Yous et al. [14]	*OMIs of QoL and QoD*<br>• QUALID<br>*OMIs for clinical outcomes*<br>• PACSLAC-II<br>• NPI-NH<br>*Other OMIs*<br>• FPCR<br>• FAVS-D	Not included	*Residents:*<br>• QUALID: no statistically significant changes<br>• PACSLAC-II: no significant changes in both unadjusted and adjusted models<br>• NPI-NH: no significant changes at all time points<br>− adjusted model: Significant ↑ at 3-month mark (95% CI) -4.89 (-9.39, -0.39)<br>− more neuropsychiatric symptoms in females than males 5.64 (1.16, 10.11)<br>*Family caregivers*:<br>• FPCR:<br>− significant ↑ at 3-month, unadjusted: -25.63 (-45.90, -6.36) and 6-month, unadjusted: −31.23 (-54.10, -8.31)<br>− similar ↑ in adjusted model at 3-month -19.57 (-37.40, -1.80) and 6-month -25.52 (-48.90, -2.09)<br>• FAVS-D: statistically significant (95% CI)<br>− adjusted model: ↓ scores with longer resident stay, indicating improved perceptions of caregiving -3.71 (-6.57, -0.85)<br>− better perceptions by family carers of female residents than male residents 37.15 (11.86, 62.40)	*Residents:*<br>• ↓ agitation and negative behavioral responses

Yous et al. [29]	Not included	Not included	Not included	*Residents:*<br>• ↑ quality of life<br>• ↑ communication and verbalization<br>• ↑ positive emotional responses (smiling, laughing)<br>• ↓ responsive behaviors/agitation<br>• ↑ calmness and relaxation<br>• ↑ alertness and awareness of surroundings<br>*Staff:*<br>• ↓ responsive behaviors requiring medication<br>• ↑ relationships with residents<br>• ↑ time for meaningful engagement<br>*Volunteers:*<br>• ↑ knowledge and skills in engaging people with advanced dementia<br>• ↑ confidence and sense of purpose<br>*Family carers:*<br>• ↑ quality of visits<br>• ↑ meaningful engagement with relatives<br>• ↑ involvement in care<br>• ↑ satisfaction and reassurance
Yous et al. [30]	*OMIs of QoL and QoD*<br>• QUALID<br>*OMIs for clinical outcomes*<br>• PACSLAC-II<br>• NPI-NH<br>*Other OMIs*<br>• FPCR<br>• FAVS-D	Not included	*OMIs of QoL and QoD*<br>• QUALID: no statistically significant change over time<br>*OMIs for clinical outcomes*<br>• PACSLAC-II: no statistically significant change over time<br>• NPI-NH: no statistically significant change over time<br>*Other OMIs*<br>• FPCR: statistically significant ↓ in family carer role stress<br>− parameter estimate = −2.11 (95% CI −2.79 to −1.44), $p<0.001$<br>• FAVS-D: no significant change	*Residents:*<br>• ↑ positive emotional responses (joy, calmness, engagement)<br>• ↓ agitation and responsive behaviors<br>• ↑ participation in meaningful, individualized activities<br>• ↑ overall well-being<br>*Family caregivers:*<br>• ↑ laughter and communication during visits<br>• ↑ visit quality<br>• ↑ perceived well-being of their relative<br>• ↑ satisfaction with care<br>*Staff and volunteers:*<br>• ↑ observed resident joy and meaningful interaction<br>• ↑ job satisfaction<br>• ↑ support for person-centered, comfort-focused care

				• ↑ sense of purpose and positive reinforcement
Yous et al. [31]	Not included	Not included	Not included	*Caregivers:*<br>• ↑ positive caregiving experiences<br>• ↑ skills and awareness<br>• ↑ eagerness to use Namaste Care<br>• ↑ learning, empowerment, and peer support<br>• ↑ feeling supported and respected in research involvement<br>• ↑ valued caregiver contributions

Source: The authors; Note: ↑ = increase, ↓= decrease, → = implicate, $\bar{x}$ = mean, $\bar{d}$ = mean difference, r = correlation index, t = t-statistics, p = p - value, level of significance, CI = confidence interval

Supplemental Material 1: Scores of the Mixed Methods Appraisal Tool (MMAT)

Study	Quality / Score	S1.	S2.	1.1. 2.1.	1.2. 2.2.	1.3. 2.3.	1.4. 2.4.	1.5. 2.5.	3.1. 4.1.	3.2. 4.2.	3.3. 4.3.	3.4. 4.4.	3.5. 4.5.	5.1.	5.2.	5.3.	5.4.	5.5.
Bray et al. [20]	60%	1	1						1	1	0	1	0					
Bray et al. [21]	80%	1	1						1	1	0	1	1					
Bray et al. [22]	80%	1	1	1	1	1	1	0										
Chang et al. [37]	80%	1	1	1	1	1	1	0										
Dalkin et al. [10]	80%	1	1	1	1	1	1	0										
De Vocht et al. [32]	80%	1	1											1	1	1	0	1
El Alili et al. [5]	80%	1	1	1	1	1	0	1										
Froggatt et al. [24]	80%	1	1	1	1	1	1	0										
Haaksma et al. [11]	100%	1	1	1	1	1	1	1										
Kaasalainen et al. [27]	80%	1	1	1	1	1	1	0										
Kaasalainen et al. [28]	100%	1	1											1	1	1	1	1
Karacsony et al. [39]	80%	1	1											1	1	1	0	1
Karacsony et al. [40]	60%	1	1											1	1	1	0	0
Kohne et al. [9]	80%	1	1						1	1	0	1	1					
Latham et al. [23]	80%	1	1											1	1	1	0	1
Li et al. [6]	100%	1	1	1	1	1	1	1										
McNiel and Westphal [35]	100%	1	1	1	1	1	1	1										
Nicholls et al. [38]	80%	1	1	1	1	1	1	0										
Simard and Volicer [36]	60%	1	1						1	1	0	0	1					
Smaling et al. [2]	100%	1	1	1	1	1	1	1										
Smaling et al. [34]	100%	1	1	1	1	1	1	1										
St John and Koffman [8]	80%	1	1	1	1	1	1	0										
Stacpoole et al. [25]	80%	1	1						1	1	0	1	1					
Stacpoole et al. [26]	80%	1	1	1	1	1	1	0										
Tasseron-Dries et al. [33]	80%	1	1	1	1	0	1	1										
Yous et al. [12]	100%	1	1											1	1	1	1	1
Yous et al. [13]	80%	1	1	1	1	0	1	1										
Yous et al. [14]	80%	1	1						1	1	1	0	1					
Yous et al. [29]	100%	1	1	1	1	1	1	1										
Yous et al. [30]	100%	1	1											1	1	1	1	1
Yous et al. [31]	100%	1	1	1	1	1	1	1										

Note: 1 yes, 0 no, NA can´t tell, Quality/Score represents the percentage of design-specific MMAT criteria rated "Yes" (Yes ÷ applicable criteria × 100). Screening questions (S1, S2) were not included in score calculation. Although the developers of MMAT discourage reliance on a single overall score, percentages are presented for transparency and comparability across studies, alongside criterion-level ratings. Source: The authors

Supplemental Material 2: Scores of the Consolidated Health Economic Evaluation Reporting Standards (CHEERS)

CHEERS section/item	Item No	Bray et al. [21]	Bray et al. [20]	El Alili et al. [5]	Froggatt et al. [24]	Kaasalainen et al. [28]
**Title and abstract**						
Title	1	Y	Y	Y	Y	Y
Abstract	2	Y	Y	Y	Y	Y
**Introduction**						
Background and objectives	3	Y	Y	Y	Y	Y
**Methods**						
Health economic analysis plan	4	Y	Y	Y	Y	P
Study population	5	Y	Y	Y	Y	Y
Setting and location	6	Y	Y	Y	Y	Y
Comparators	7	Y	Y	Y	Y	N
Perspective	8	Y	Y	Y	Y	N
Time horizon	9	Y	Y	Y	Y	Y
Discount rate	10	NA	NA	NA	NA	NA
Selection of outcomes	11	NA	Y	Y	Y	Y
Measurement of outcomes	12	NA	Y	Y	Y	Y
Valuation of outcomes	13	NA	Y	Y	Y	P
Measurement and valuation of resources and costs	14	Y	Y	Y	Y	Y
Currency, price date, and conversion	15	Y	Y	Y	Y	P
Rationale and description of the model	16	Y	Y	Y	Y	Y
Analytics and assumptions	17	Y	Y	Y	Y	Y
Characterizing heterogeneity	18	NA	N	N	Y	Y
Characterizing distributional effects	19	Y	Y	Y	Y	Y
Characterizing uncertainty	20	NA	Y	Y	P	N
Approach to engagement with patients and others	21	NA	NA	Y	Y	Y
**Results**						
Study parameters	22	Y	Y	Y	Y	Y
Summary of main results	23	Y	Y	Y	Y	Y
Effect of uncertainty	24	NA	Y	Y	Y	Y
Effect of engagement with patients and others	25	NA	Y	Y	Y	Y
**Discussions**						
Study findings, limitations, generalizability, and current knowledge	26	Y	Y	Y	Y	Y
**Other**						
Source of funding	27	Y	Y	Y	Y	Y

Conflict of interest	28	Y	Y	Y	Y	Y
**Reporting quality based on % score**		100.0%<br>Strong	95.8%<br>Strong	96.3%<br>Strong	100.0%<br>Strong	82.7%<br>Moderately strong

Note: Y – yes P – partially reported, N – not reported, NA – not applicable, Points: Y = 1, P = 0.5, N = 0, percentage score was calculated after the exclusion of "not applicable" item (Del Pino et al., 2022).
Source: The authors
