# Supplementary material for: Exploring the impact of Namaste Care for individuals with advanced dementia: a systematic review of costs, effects and benefits
Source: BMJ Open. 2026 May 6;16(5):e102899. doi: 10.1136/bmjopen-2025-102899 (PMC13150879; doi:10.1136/bmjopen-2025-102899)
Supplement: Supplementary file 2 [file bmjopen-2025-102899.reviewer_comments.pdf]

PEER REVIEW HISTORY

BMJ Open publishes all reviews undertaken for accepted manuscripts. Reviewers are asked to complete a checklist review form and are provided with free text boxes to elaborate on their assessment. These free text comments are reproduced below.

ARTICLE DETAILS

Title (Provisional)

Exploring the Impact of Namaste Care for Individuals with Advanced Dementia: A Systematic Review of Costs, Effects and Benefits

Authors

Parks, Tereza; Soukopová, Jana; Tóthová, Dominika; Smaling, Hanneke J.A.; Murphy , Aileen; Vidovićová, Lucie; Hlavka, Jakub

---

VERSION 1 - REVIEW

**Reviewer**	**1**
**Name**	**Fukuda, Yoshiharu**
**Affiliation**	**Teikyo University Graduate School of Public Health, Community Health and Medicine**
**Date**	**12-Jun-2025**
**COI**	**None**

The topic is important and the review was conducted in standard manner. And the manuscript was written suitably for publication.

**Reviewer**	**2**
**Name**	**Kelson, Zoe**
**Affiliation**	**University of Exeter, Mathematics**
**Date**	**06-Oct-2025**
**COI**	**None**

This systematic review explores the intervention Namaste Care and its clinical and economic effects in multiple care settings. The aim of this review is to consolidate existing evidence on Namaste Care's clinical and economic outcomes and examine the tools used for data collection.

Reviewer comments:

"PROSPERO; registration number CRD42024560056"

The study protocol has been suitably registered by the authors.

"We report our findings in accordance with the PRISMA statement [16] and the PRISMA-COSMIN for Outcome Measurement Instruments [17]."

Thanks for providing a copy of the PRISMA checklist.

"A systematic literature search was conducted (pubmed, Scopus, and Web of Science) to identify peer-reviewed studies on Namaste Care's impact on quality of life, costs, health, economic outcomes, and benefits (2010-2024)."

Can the search dates please be specified in the Abstract?

"A systematic literature search was performed in the PubMed, Scopus, and Web of Science databases between June 21 and July 18, 2024. "

Can the literature search please be updated prior to publication?

"the search was limited to English-language"

Can the authors please consider utilising a translation tool to include articles published in other languages?

"studies published between January 1, 2010, and June 21, 2024. The time span was chosen because of the publication of a seminal paper on Namaste Care [18] and the growing rate of the intervention's implementation in the late 2010s."

Can the timespan please be opened up (i.e. to before 2010) to include more articles?

A subgoup analyses by date of publication can then be conducted, if required?

"The titles and abstracts of all the papers retrieved through the search strategy were initially independently screened for relevance by two reviewers (TP and JS)...

Three team members (TP, JS, DT) independently assessed the full-text papers. Each reviewer evaluated whether each study met the eligibility criteria. In cases of disagreement, the reviewers engaged in a discussion to resolve the differences. If the discussion failed to reach a consensus, a fourth reviewer (JH) was consulted for the final decision"

and

"At least four members of the six members of the review team (TP, JS, DT, JH, LV, HJAS) independently assessed all the extracted data in all the studies to ensure thoroughness and accuracy. Any disagreements or discrepancies in the extracted data were resolved through discussion between at least two review team members to reach a consensus."

The authors demonstrate that they have followed rigorous article selection and data extraction processes.

"Study quality was assessed via the Mixed Methods Appraisal and Consolidated Health Economic Evaluation Reporting Standards 2022" [Abstract]

and

"To assess the methodological quality of the included studies, we used the Mixed Methods Appraisal Tool (MMAT) [19] for assessing quantitative, qualitative, and mixed methods studies and the Consolidated Health Economic Evaluation Reporting Standards 2022 (CHEERS) [20] for economic evaluation studies. Each study was independently evaluated by a team of three reviewers (TP, JS, DT). All disagreements in quality ratings were resolved by consensus. "

The authors have suitably assessed study quality.

"The results of the MMAT are shown in Supplemental Data Section 1, and the results of the CHEERS are shown Supplemental Data Section 2. "

Can the authors please comment on how study inferences may be impacted if articles deemed to be of low or moderate quality are excluded (e.g. exploring a qualittative subgroup analysis exploring the robustness of the study findings to study quality)?

Can the authors please communicate their rationale for not performing a meta-analysis in this study?

"• The review was limited to peer-reviewed, English-language publications that explicitly referenced Namaste Care, which may have excluded relevant studies published in other languages or under different terminology. • The short duration of the included studies restricts the ability to draw conclusions about the long-term effectiveness and cost implications of the intervention for individuals with dementia, caregivers, and care staff. " [Strengths and Limitations of this Study]

Can the study limitations please also be expanded on in the Discussion section?

VERSION 1 - AUTHOR RESPONSE

Reviewer 1

**Comment R1.1**<br><br>**“The topic is important and the review was conducted in a standard manner. And the manuscript was written suitably for publication.”**<br><br>**Response:**<br>We thank the reviewer for this positive assessment of our work. We appreciate the acknowledgement that the review was conducted in a standard manner. No changes were required in response to this comment.

Reviewer 2

Comment R2.1

“Can the search dates please be specified in the Abstract?”

Response:
Thank you for this helpful comment. We have added the exact search dates to the Abstract.

Manuscript changes:
Abstract.

Abstract: “*A systematic literature search was conducted (PubMed, Scopus, and Web of Science) to identify peer-reviewed studies on Namaste Care’s impact on quality of life, costs, health, economic outcomes, and benefits up to February 22, 2026*”

Comment R2.2

“Can the literature search please be updated prior to publication?”

Response:
We thank the reviewer for this suggestion. We have rerun the literature search using the same databases and search strategy as originally specified, including studies published up to February 22, 2026. The Results section, PRISMA flow diagram, and Supplementary Materials have been updated accordingly.

Manuscript changes:
Methods, Results, PRISMA flow diagram.

Comment R2.3

“Can the authors please consider utilising a translation tool to include articles published in other languages?”

Response:
We thank the reviewer for this thoughtful suggestion. We carefully considered the inclusion of non-English studies. However, due to resource constraints and the need to ensure accurate interpretation of clinical and economic outcomes, we retained the English-language restriction as specified in the eligibility criteria.

In the updated search conducted without language restrictions, we identified only one additional article published in Persian. Given the potential risk of misinterpretation when using automated translation tools for complex clinical and economic data, we decided not to include this study. This limitation is now more explicitly acknowledged and discussed in the Discussion section.

Manuscript changes:
Discussion, Limitations.

Comment R2.4

"Can the timespan please be opened up (i.e. to before 2010) to include more articles?"
"A subgroup analyses by date of publication can then be conducted, if required?"

Response:
Thank you for this helpful suggestion. The search timespan has now been extended to include studies published before 2010. The Methods section and the Search Strategy subsection have been updated accordingly to reflect this change.
With regard to conducting subgroup analyses by date of publication, we considered this approach; however, due to the substantial heterogeneity in study designs, outcome measures, and reporting formats across the included studies, as well as the limited number of studies within individual time periods, we determined that such subgroup analyses would be unlikely to yield meaningful or robust comparisons. In addition, the primary aim of this review was to provide a comprehensive descriptive synthesis of the evidence rather than to examine temporal trends.

Manuscript changes:
Methods, Search Strategy.

Comment R2.5

"The authors demonstrate that they have followed rigorous article selection and data extraction processes"
"The authors have suitably assessed study quality."

Response: We thank the reviewer for this positive assessment of our work. We appreciate the acknowledgement that the review was conducted in a standard manner. No changes were required in response to this comment.

Comment R2.6

"Can the authors please comment on how study inferences may be impacted if low- or moderate-quality studies are excluded?"

Response:
We thank the reviewer for this important question. We have revised the quality appraisal table to align more closely with the principles of the Mixed Methods Appraisal Tool (MMAT). Specifically, we removed the previously used overall quality labels (e.g., *strong*, *moderately strong*, *moderately weak*), as the MMAT does not generate numerical quality categories or recommend percentage cut-offs (http://mixedmethodsappraisaltoolpublic.pbworks.com/w/page/71030694). Instead, we use the MMAT to support a narrative appraisal of methodological strengths and limitations rather than to apply strict quality thresholds. For transparency, we report the proportion of MMAT criteria met by each study (Supplemental Data Section 1).

Three studies (Bray et al. [21], Karacsony et al. [30], and Simard and Volicer [18]) met approximately 60% of the applicable MMAT criteria, while all remaining studies met 80% or 100% of criteria. Excluding these lower-scoring studies would have a very limited impact on the overall findings. Restricting the synthesis to studies with the strongest methodological designs would substantially

reduce the evidence base, leaving primarily the one randomised controlled trial (Smaling et al. [34]) and a small number of mixed-methods and qualitative studies.

This reflects a deliberate, protocol-specified choice to include all eligible studies regardless of quality in order to comprehensively map the available evidence, with quality appraisal used to aid interpretation rather than as an exclusion criterion. Importantly, when considering only higher-quality studies, the main inferences remain unchanged: evidence of statistically significant improvements in quality of life is limited; cost data suggest modest increases in resource use with potential medication savings; and qualitative evidence consistently indicates benefits for residents and caregivers. However, restricting inclusion to higher-quality studies would reduce the breadth of qualitative and implementation evidence.

We have clarified this rationale in the Methods and Discussion sections and added a paragraph describing how inferences would change under a quality-threshold restriction.

Manuscript changes:

Methods: *"To assess the methodological quality of the included studies, we used the Mixed Methods Appraisal Tool (MMAT) [19] for quantitative, qualitative, and mixed-methods studies. For economic evaluation studies, the Consolidated Health Economic Evaluation Reporting Standards 2022 (CHEERS) were used to assess the completeness of reporting. Each study was independently evaluated by a team of three reviewers (TP, JS, DT). All disagreements in ratings were resolved by consensus. The results of the MMAT are presented in Supplemental Data Section 1, and the results of the CHEERS assessment are presented in Supplemental Data Section 2."*

Discussion: "*A further limitation concerns the inclusion of studies regardless of methodological quality. This was a deliberate protocol-specified decision to ensure comprehensive mapping of the evidence. In our assessment (MMAT, Supplemental Data Section 1), we indicate that only three studies [18, 21, 30] met approximately 60% of the applicable MMAT criteria, while all remaining studies met 80% or 100% of the criteria, which has a very limited impact on the overall findings of our study. To address the potential impact of this approach on study inferences, we conducted a sensitivity analysis restricted to higher-quality studies — primarily the three randomized controlled trials Froggatt et al. [41] and El Alili et al. [5], Smaling et al. [34] and the stronger mixed-methods studies. The core directional conclusions of the review remained unchanged: statistically significant quality-of-life benefits were limited, cost data indicated modest increases in resource use with potential medication savings, and qualitative benefits to residents and caregivers were consistently reported. However, restricting to higher-quality studies substantially narrows the evidence base, as much of the implementation and experience literature derives from feasibility and pilot studies. This further underscores the need for larger, methodologically rigorous trials."*

Comment R2.7

"Can the authors please communicate their rationale for not performing a meta-analysis?"

Response:

We thank you for this question. A meta-analysis was not undertaken because the available evidence did not provide sufficient and comparable quantitative data to support a meaningful pooled analysis. Many of the included original studies reported outcomes descriptively, used non-uniform

measurement instruments, or lacked the statistical parameters (e.g. effect sizes, variance measures) required for quantitative synthesis.
Given that the primary aim of this review was to identify and synthesise original studies evaluating the clinical and economic outcomes of Namaste Care, as well as to examine the range of outcome measures and data collection tools used, a systematic review was considered the most appropriate and methodologically robust approach.

Manuscript changes:
None required.

Comment R2.8

"Can the study limitations please also be expanded on in the Discussion section?"

Response:
Thank you for this comment. We have expanded the Discussion section to include a more detailed and structured limitations subsection. This now explicitly addresses the language restriction and acknowledges the fact that heterogeneity in the quality of studies included affects the strength of the evidence base to date. As factors outside of our control, we provide this as additional context for readers, and further underscore the need for larger, methodologically rigorous trials.

Manuscript changes:
Discussion
